# Marek’s disease in chickens: a review with focus on immunology

**DOI:** 10.1186/s13567-016-0404-3

**Published:** 2016-11-28

**Authors:** Nitish Boodhoo, Angila Gurung, Shayan Sharif, Shahriar Behboudi

**Affiliations:** 1 The Pirbright Institute, Ash Road, Pirbright, Woking, Surrey, GU24 0NF UK; 2 Department of Pathobiology, Ontario Veterinary College, University of Guelph, Guelph, ON Canada

## Abstract

Marek’s disease (MD), caused by Marek’s disease virus (MDV), is a commercially important neoplastic disease of poultry which is only controlled by mass vaccination. Importantly, vaccines that can provide sterile immunity and inhibit virus transmission are lacking; such that vaccines are only capable of preventing neuropathy, oncogenic disease and immunosuppression, but are unable to prevent MDV transmission or infection, leading to emergence of increasingly virulent pathotypes. Hence, to address these issues, developing more efficacious vaccines that induce sterile immunity have become one of the important research goals for avian immunologists today. MDV shares very close genomic functional and structural characteristics to most mammalian herpes viruses such as herpes simplex virus (HSV). MD also provides an excellent T cell lymphoma model for gaining insights into other herpesvirus-induced oncogenesis in mammals and birds. For these reasons, we need to develop an in-depth knowledge and understanding of the host-viral interaction and host immunity against MD. Similarly, the underlying genetic variation within different chicken lines has a major impact on the outcome of infection. In this review article, we aim to investigate the pathogenesis of MDV infection, host immunity to MD and discuss areas of research that need to be further explored.

## Introduction

**Figure 1:**
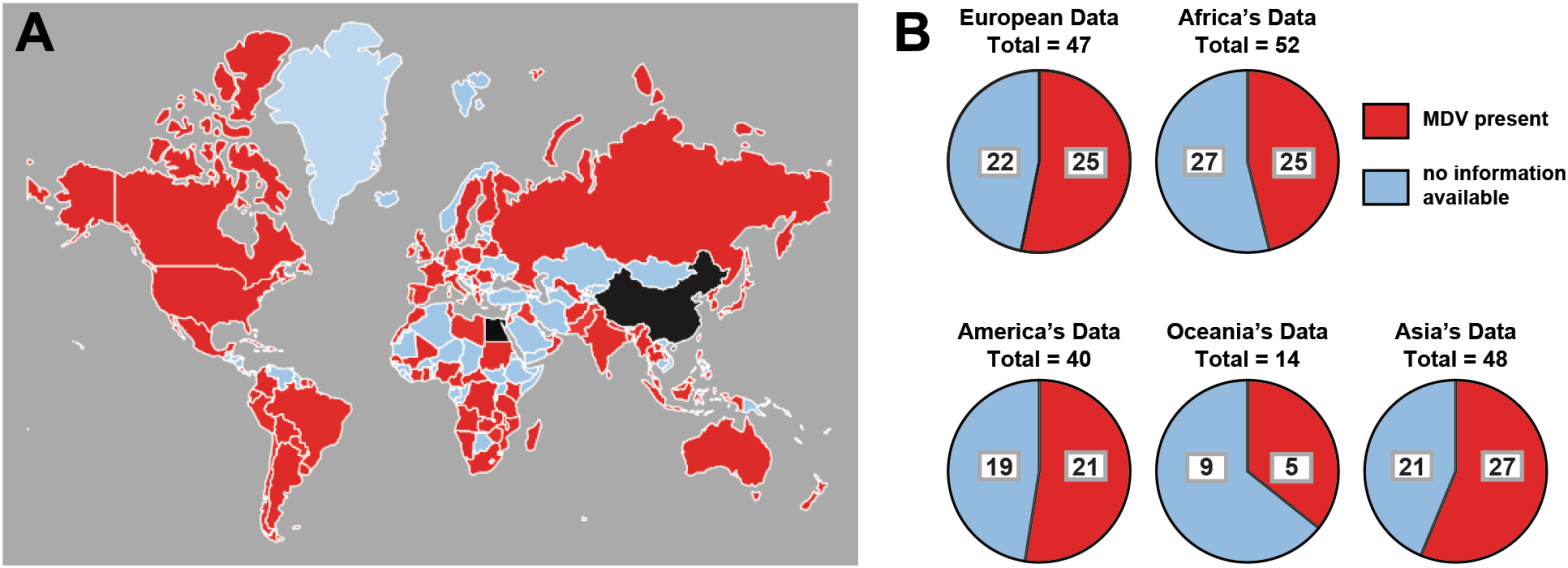
**Worldwide map depicting distribution of MDV whereby evidence for presence has been submitted to the OIE.** Distribution data was obtained from the World Organization for Animal Health (OIE) distributed through the World Animal Health Information Database (WAHIS) interface and summarized above based on absence (blue) and presence of disease with reported cases (red) before and after 2009. **A** World map depicting countries positive for MDV was constructed using the imapbuilder software. Both China and Egypt (black) are endemic areas for MDV with outbreaks reported on a yearly basis. **B** Pie chart demonstrates the number of countries that have reported MDV cases to the OIE based on their geographical location (continent). Data is summarized based on absence (blue), and presence of disease with reported cases (red).

Characterized after its human orthologue (Herpes Simplex Virus; HSV a DNA containing virus), Marek’s disease virus (MDV), or Gallid herpesvirus 2 (GaHV-2), the etiologic agent for Marek’s disease (MD) is an *alpha*-herpes virus that targets avian species (*Gallus gallus domesticus*) where it establishes chronic infection. Notably recognized as a multifaceted disease, MD is characterized based on immunosuppression, neurological disorders and neoplastic transformation of CD4+ T cells, localised around peripheral nerves and visceral organs of the host [1]. Since the late 1960s, both large and small scale poultry production systems have been dependent on vaccine use for disease control. Although not a notifiable disease according to the World Organization for animal health (OIE), disease distribution is acknowledged as worldwide although precise estimates of morbidity, annual economic loses and report of disease distribution on each continent are lacking. Effective global surveillance for MDV requires accuracy of reporting source and comprehensiveness. Current data from OIE estimates that about half of the world countries have reported cases of MDV infection (Figure 1). As global requirement increases, so does our dependence on intensive poultry production facilities. The control of MDV infection is arguably very challenging due to its ubiquitous presence at the expense of already pre-established biosecurity programs.

Several MDV pathotypes have been characterised over the years based on morbidity and mortality rates [2]. These pathotypes can be distinguished into three subfamilies (GaHV-2, GaHV-3 and MeHV-1) based on biological and genomic similarities: GaHV-2 (RB-1B, Md5 and CVI988/RISPENS), GaHV-3 (SB-1) and MeHV-1 (HVT; FC-126). Like all herpes viruses, MDV is strictly cell associated. MeHV-1 also known as the Herpes Virus of Turkey (HVT/FC-126) is non-pathogenic in chickens and turkeys [3], but can induce viremia, which is associated with the induction of protective immunity against MD. Chickens infected with HVT become persistently infected and maintain long-lasting immunity. Comparative genomic analysis identified sequence similarities, features and structures to that of infectious MDV (GaHV-2) antigens [4]. Live HVT (FC-126 strain) in combination with SB-1 and 301B/1 are widely used in bivalent and polyvalent vaccine formulation to provide enhanced protection against GaHV-2, but they became less effective with the emergence of highly virulent MDV. The most effective vaccines developed to date is CVI988/RISPENS (GaHV-2) vaccines [5] which provides protective immunity against disease progression (oncogenesis and neuropathy; transient and acute paralysis) but not sterile immunity (establishment of primary infection and shedding of mature virus particle) which is complicated by reactivation of virus particles from latency. Since its introduction, global vaccination efforts have been efficacious in controlling disease outbreaks thus increasing poultry operations productivity and reduced losses associated with large scale culling of diseased chickens. MD is the first oncogenic disease for which an effective vaccine has been developed. New surging evidence suggests that current vaccination protocol, whether imperfect [6] may be acting in synergy with a plethora of environmental factors resulting in MDV genetic drifts [7–9]. The human *alpha*-herpes virus, HSV and Varicella Zoster Virus (VZV) [10] are well documented with similar pathogenesis to MDV. The Oka vaccine provides highly protective immunity against VZV primary infection (chicken pox) and controls viral dissemination (virus reactivation from latency in nervous system cells), unlike the CVI988/RISPENS strain. In MD, although clinical onset of disease has been controlled with implementation of vaccination programs, MDV continues to infect and replicate into fully infectious virus particles in vaccinated chickens; followed by the shedding of these highly contagious, cell-free mature virions from the feather follicle epithelium into skin dander and poultry dust. All these make MDV environmentally persistent as well as a highly infectious and contagious pathogen of chickens. In this review article, we discuss recent progress made in understanding MDV pathogenesis and immunity, aim to scrutinize the pathogenesis of MDV infection, host’s immunity to MD and outline areas of research that need to be further explored.

## Pathogenesis of Marek’s disease

### Marek’s disease

**Figure 2:**
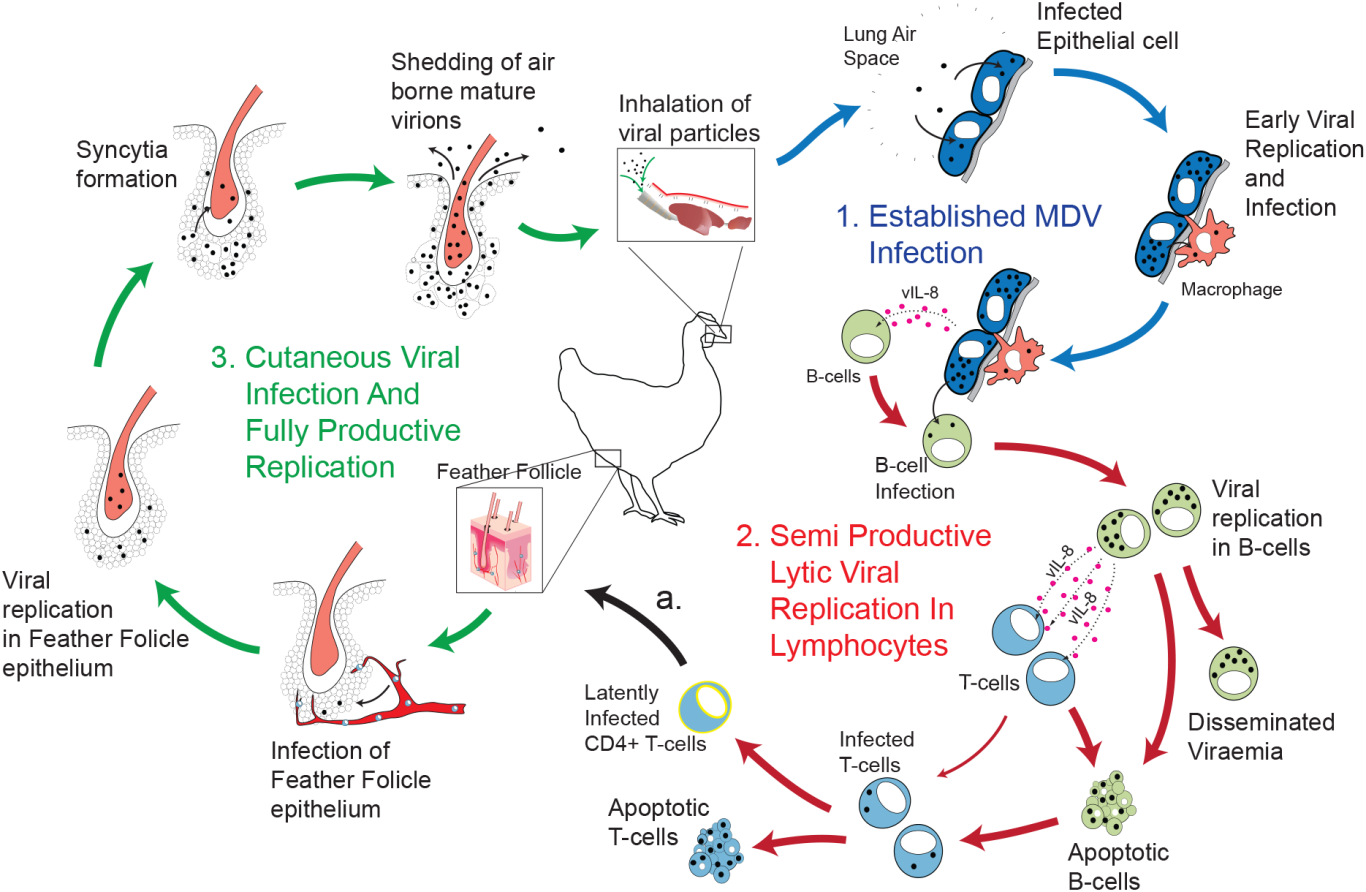
**Model of MDV infectious life cycle in resistant birds.** MDV infection of naive host occurs via inhalation of dust or skin dander encapsulated viral particles into the respiratory tract. *1* Primary infection occurs when virus particle breaks mucosal tolerance in the lungs, site of entry into the epithelial cells. Local viral replication establishes infection and initiates viral immediate-early gene, viral Interleukin-8 (vIL-8), transcription and translation. Inflammatory responses in the underlying tissue recruit innate immune system cells which result in uptake of infectious virus particle by macrophages. Infiltration of lymphocytes via action of vIL-8 follows resulting in MDV infection of B-cells. *2* Viral replication in B cells initiates Semi Production Lytic Viral Infection and disease progression. MDV infected B cells secret vIL-8 that acts as a chemotactic factor for and gains access to T-cells. This specific lymphotropism (B cells and T cells) enables systemic disseminated viraemia. Viral replication causes apoptosis of B and T lymphocytes in a hallmark of immunosuppression. MDV integrates specifically into the genome of CD4+ T cells enabling escape from immune detection and initiates Latent Viral Infection. Early latently infected and activated CD4+ T cells have not been phenotypically characterised by cell surface markers. *a* Early latently infected and activated CD4+ T cells migrate to cutaneous sites of replication namely feather follicle. *3* Infection of feather follicle epithelium enables fully productive viral replication. Viral replication results in syncytia formation. Infection of feather epithelium leads to secretion of mature virion in skin danders and dust that act as the major source of infectious materials. Horizontal transmission is the only recognized form for environmental persistence and infection in field conditions. Systemic infection and neoplastic transformation of CD4+ T cells in susceptible birds is further discussed (Figure 3).

MDV, the causative agent of MD in chickens, result in transformation of CD4+ T cells. The natural route of infection is defined by inhalation of airborne cell-free virus particles within the contaminated dust and dander (Figure 2), shed from infected host produced in terminally differentiated feather follicle epithelium [11], into a naïve respiratory track [12]. MD pathogenesis has four phases in the susceptible birds; an early cytolytic phase within 2–7 days post-infection (dpi) which delineates as semi productive lytic viral replication in lymphocytes. This is followed by a latency phase that occurs between 7 and 10 dpi in CD4+ T cell subset that result in systemic viral dissemination. Cutaneous viral infection can occurs as early as 4 dpi and eventually results in fully productive viral replication and shedding [13]. MDV reactivation in CD4+ T cells initiates a late cytolytic and immunosuppressive phase starting around 18 dpi. Finally a proliferative phase around 28 dpi [14, 15] is characterized by formation of visceral tumours that originate from CD4+ T cells lymphoma. There is no transmission from chicken to eggs (vertical transmission) but the birds are usually infected in early stage after hatching (horizontal transmission). The presence of maternal antibody against MD can protect the neonatal chicks, and with the development of a functional immune system a degree of resistant to MD is developed [5]. However, husbandry-related stress or concurrent infection with other immunosuppressive pathogens such as chicken infectious anaemia virus (CIAV), reovirus and infectious bursal disease virus (IBDV), significantly enhances susceptibility to MDV [5]. Another important factor in the susceptibility to MD is the genetic background of chicken lines which is to some extent associated with the major histocompatibility complex (MHC). However, it should be noted that MHC does not necessarily play a critical role in resistance or susceptibility to MD. Chicken lines 6-3 and 7-2 harbour the same MHC haplotype (B2/B2) while line 6-3 is relatively resistant and line 7-2 is highly susceptible to MD. It has been demonstrated that some very virulent (vv+) strains of MDV induces tumours even in the resistant line 6-3. MDV-associated lymphoma can only be developed in genetically susceptible chickens; however the virus can replicate and shedding still occurs in both susceptible and resistant chicken lines.

### Establishment of primary infection

It is speculated that lung epithelial cells are one of the primary target cells for MDV infection. *MDV* antigens, with well-defined expression during cytolytic and latent phase of replication, have been detected at significant levels at various time points in lung epithelial cells in ovo [16], and in vivo [17] suggesting an establishment of successful infection. The later was performed via an aerosol method which simulates natural infection as a respiratory disease [12]. Viral replication in the lungs could be detected as early as 1 dpi. Purchase et al. [18] were among the first to demonstrate a novel route for high replication kinetics of infectious MDV antigens in lungs epithelial cells of chicks inoculated via intra-abdominal route. However when they repeated the experiment, a lower immunofluorescence was detected at 5 dpi compared to 7 dpi. The route of administration, whether intra-abdominal or intra-tracheal might affect viral replication as well as systemic dissemination that results in MD [19]. In addition, infection of lung resident antigen presenting cells (APCs), such as macrophages, is thought to result in subsequent transport to primary and secondary lymphoid organs such as thymus, bursa of fabricius, and spleen [20]. Although it is unclear whether macrophages and lung epithelial cells get infected simultaneously or rather infected lung epithelial cells may play a role in transmitting viral particles to macrophages. It is evident that post MDV infection, immune responsiveness leads to macrophage infiltration although viral replication is unaffected [17]. It is also believed that presence of MDV particles in the lung, during the earliest infection, stimulates secretion of cytokines and chemotactic factors that help in attracting B cells to site of infection [21, 22]. One of the defined chemokine is a viral IL-8 which is similar to CXCL13 and is involved in recruiting immune systems cells to site of viral replication [23] and is defined as a homologue to the host IL-8 gene. IL-8 has a well-defined role as a chemotactic molecule for T cell [22, 24] and B cells [22]. Immune cells recruited to the lung such as B cells can be detected as early as 2 dpi [25].

### Semi productive lytic viral replication

MDV has a specific tropism for immune system cells and preferentially infects lymphocytes; B cells and T cells (αβ). Infection of B cells may occur in the lung and viral replication in B cells is defined as semi-productive lytic viral replication. Lytic activity due to viral replication has been linked to phosphoprotein 38 (pp38) activities [26, 27]. PP38 role as an early immediate gene is defined only in lymphocytes, specifically B cells and T cells [28, 29]. It has been shown that an MDV rMd5Deltapp38 deletion mutant for pp38 lacked the ability to induce cytolytic activity characterized by B cell apoptosis [30]. This is in accordance with the notion that recruitment of lymphocytes such as B cells to site of viral replication is a key step for transmission of virus and dissemination. Deletion mutants of vIL-8 (RB1BvIL-8ΔsmGFP) when tested in vivo showed a reduced capacity to successfully infect lymphocytes and induce lytic infection [23]. A lack of IL-8 therefore result in impaired ability to recruit B cells and as well as an observable reduction in cytolytic activity due to reduced viral titer and dissemination by lymphocytes. Infection with wild-type MDV restored lytic activity and viral dissemination to primary lymphoid organs [28]. Viral lytic activity in B cells results in a drastic downfall in the overall antibody production and ability to fight against an infection. Consequently, the infected chicken suffers from immunosuppression, making it more susceptible to MD and other infections [31]. During the early cytolytic phase, transcriptional modification and epigenetic changes (DNA methylation, histone post-translational modifications and non-coding RNAs), along with post-transcriptional and post-translational modifications, regulate viral replication cycle and subsequent expression of cellular and viral genes. Either way, it’s been postulated that disseminated viremia to various organs in a cell associated manner results in systemic infection. Infected B cells may also be able to produce vIL-8 mRNA which would have chemotactic activity for T cells. Infected B cells therefore are able to recruit T cells which would allow for transmission of MDV virus particle from infected B cells to activated T cells. Viral replication in T cells also lead to cytolysis associated with pp38 activity. The RB1BvIL-8ΔsmGFP deletion mutant lacked the ability to attract B cells and subsequently impaired its ability to infect T cells albeit at a lower frequency [23]. Furthermore, MDV preferentially targets CD4+ T cell subsets and infection results in viral latency and immune evasion [22].

### Immune evasion and latency

**Figure 3:**
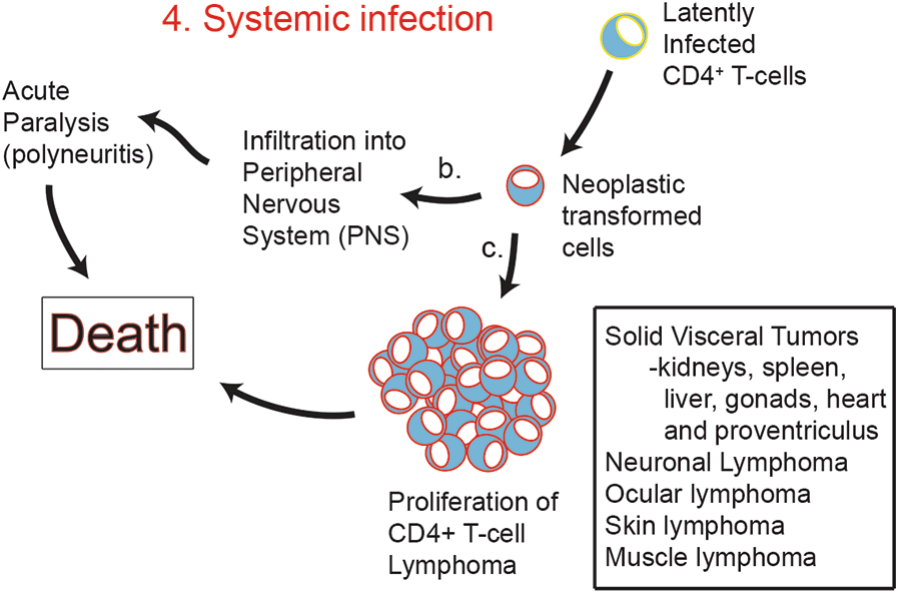
**Model of MDV neoplastic transformed CD4+** **T cells and subsequent disseminated Systemic Infection in susceptible birds.** *4* Fully latent MDV-Transformed CD4+ T-cells proliferate in all sites where immune systems cells are involved in primary and secondary line of defence. Early latently activated CD4+ T cells undergo neoplastic transformation due to transcriptional and transrepressional activity of viral oncogenic proteins such as *Meq*. Pathogenesis arise in cases where vaccination failure is suspected (suboptimal dosage/titre, damage to vaccine or confirmed immunological vaccine failure), vaccination is not performed (backyard birds). *b* Fully latent neoplastic MDV-Transformed CD4+ T cells infiltrate and establish a reservoir of MDV genome in peripheral nerve fibres interspace. These cells have a CD4+ CD25+ Treg phenotype although additional cell surface markers have yet to be determined. Expression of viral neurovirulence factor, phosphoprotein 14 (pp14), promotes neuropathy and cell survival. Neuropathy (Polyneuritis) is presented as transient or acute paralysis of legs, wings, neck, with vision impairment and weight loss depending on MDV-1 virulence factor. Birds infected with serotype-1 eventually succumb to death from paralysis. *c* Reactivation from latency enables a second phase of replication whereby viral oncogenic protein *Meq* acts on T cell signaling pathways causing uncontrolled cellular proliferation leading to disseminated lymphoma formation in visceral organs, peripheral and central nervous system, musculoskeletal systems, skin and eyes. Severe lymphoma eventually causes death in birds. Highly pathogenic viruses (serotype-1, vv+ MDV) kill birds before they reach the lymphoproliferative phase of the disease.

Hereafter, MDV enters the latency phase (Figure 2) such that it can no longer be detected by the host immune system while it continues to replicate inside the lymphocytes [15]. One of the important immune evasion strategies include down regulation of MHC I molecules on infected lymphocytes. A specific gene, encoding for viral RNA telomerase (vTR) subunit, has been reported to be present only in the oncogenic MDV pathotypes, but not in their non-oncogenic counterparts. In addition, further study has confirmed about 88% identity of the vTR gene with the chicken terminal repeat (cTR) gene of the host. This would mean that vTR gene is also complement to host Telomerase reverse transcriptase (TERT) for cTR. Consequently, MDV and other herpes virus integrate at the ends of host’s chromosomes, preferably at TR sequences. It is, therefore, thought that vTR subunit might, in fact, have a significant role in maintaining the viral oncogenicity through telomeric elongations at the host’s chromosomal ends thereby inhibiting programmed cell death through telomere shortening [32]. However, it has recently been shown that both oncogenic and vaccinal strains of MDV have the ability to integrate into DNA, suggesting that integration alone is not sufficient for MDV-induced transformation [33]. Chromosomal insertions of *alpha*-herpes virus DNA segments, including those from HSV and equine herpes virus types 1 and 3, have been associated with their oncogenesis, because many of the cells carrying integrated viral DNA displayed a transformed phenotype [32]. Consistent with this, Delecluse et al. [34] has demonstrated evidences of MDV genome integration into host’s chromosomes for all lymphoma established, MDV cell lines. Additionally, expression of MDV-Meq gene is essential for MDV-induced neoplastic transformation of latently infected cells (Figure 3). Meq expressed specifically by oncogenic GaHV-2 has transcriptional activities that lends to modulation of host genomic activity. In relation to this, Lupiani et al. [35] conducted a study using a recombinant Md5 pathotype, attenuated by deletion of Meq-gene. As expected, MDV did infect and replicate in the lymphoid organs and feather follicles; but there was no tumour induction, implicating that the integrating viral genome requires a Meq gene for induction and maintenance of oncogenic properties. Reactivation of MDV from latency and tumorigenic transformation of latently infected lymphocytes mainly occurs in CD4+ T cells [36].

Another important factor in the susceptibility to MD is the genetic background of established chicken lines. MDV-associated CD4+ T cell lymphoma can only be developed in genetically susceptible chickens; however, the virus can replicate and be shed from both susceptible and resistant chicken lines. It should be noted that highly pathogenic strains of MDV can induce T cell lymphoma in the resistant chicken lines. It is believed that only a few subsets of CD4+ T lymphocytes undergo transformation, and thus are the origin of lymphoma [1]. This may explain why in most cases of MD tumour cells obtained from different anatomical sites, such as liver, kidneys, gonads, skin and muscles, all have similar CDR3 length profile suggesting that tumour cells are monoclonal [37]. In most lymphoma cells, the virus is in the latent phase and does not produce viral particles and only 0.1% of tumour cells are in the lytic phase [38]. Omar and Schat [26] have revealed an association between the genetic background of chickens and their resistance to MDV; in relation to slight variations on MDV associated-MHC presentations between resistant (B21) and susceptible (B19) chicken lines. These studies showed evidences of T cell mediated immune responses against the MDV antigen, *ICP4*, only in the resistant chicken lines; but not in the susceptible ones. In contrast, both B21 and B19 chicken lines have been reported to show T cell immune responses against the other three MDV antigens: *gB*, *Meq* and *pp38*. Nevertheless, these studies suggest that T cell mediated responses in combination with MHC play an important role in genetic resistance against MD.

### Cutaneous infection, replication and shedding

Viral genomes are detectable by quantitative PCR in blood cells and feather tips of birds infected with oncogenic or vaccinal strains [13]. Similar to other herpes viruses, MDV has a tendency to be transported towards cutaneous sites such as skin, and feather follicles. MDV is shed into the environment via scales and feather debris, which is a major source of contamination [12, 39]. It is possible that T cells transport the virus to feather follicles, but the role of other immune cells in transporting the virus has not yet been ruled out. Infiltration of CD4+ and CD8+ T cells and expression of pro-inflammatory cytokines into the skin of birds infected with a highly virulent virus (like RB-1B) or by a vaccine strain of the virus (like Rispens or HVT) suggest that the immunity against the virus is ineffective at blocking virus replication and shedding [40, 41]. Cell-free MDV is only produced in feather follicle epithelial cells, and it is believed that MDV relies exclusively on cell-to-cell transmission [42, 43]. However, in a recent report, this notion has been challenged by demonstrating that in a cell-blebbing phenomenon and cell apoptotic corps clearance mechanism, MDV can be transmitted in a cell-free condition [44]. Meq expressing tumour tissues can also be found in the skin of infected birds and neoplastic cutaneous lesions in the scaleless chickens indicates that feather follicles are not necessary for skin tumour development. Finally, the data indicate that inoculation with supernatant fluid from homogenized and sonicated skin samples of MDV-infected scaleless chickens induces MD in susceptible birds, suggesting that skin epithelial cells not associated with feather follicles also harbour infectious viral particles [45]. The process of apoptotic corps clearance may well be related to its specific tropism for adaptive immune cells which leads to immunosuppression. It is still unclear whether either phenomenon also occurs in vivo during the early hours of infection or vaccination as well as the respective cells involved. Furthermore, vaccinated challenge birds don’t show clinical signs of immunosuppression therefore an inherent resistance to cellular apoptosis is observed. This may have important implications in our understanding of MDV pathogenesis and development of next generation MDV vaccines. MDV can be detected in the feather follicles at around 11–14 dpi, using standard biochemical methods, and 6–7 dpi, using sensitive methods such as qPCR. In cutaneous sites, fully productive infection and replication is re-activated in feather-follicle epithelium and enveloped infectious viral particles in a cell free form are released. MDV can be found in the epithelium of feather follicles infected chickens more frequently than other tissues, both, in terms of incidence and levels of viral antigen expression [46]. The virus replicates as enveloped, cell free MDVs in the feather follicles epithelium of infected chickens. These cell-free MDVs are highly infectious and are easily released into the poultry dust or litter. They have a very protective envelope, allowing them to survive for months in poultry thus facilitating horizontal transmission and infection of naïve animals in following production cycles [47].

**Figure 4:**
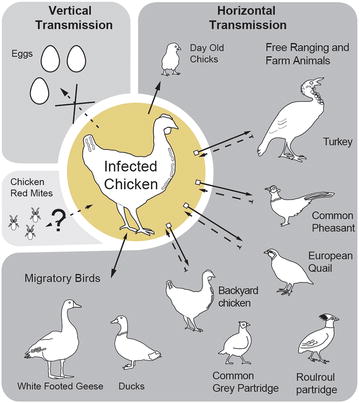
**Model for horizontal and vertical transmission of MDV between avian species.** MDV serotypes can infect several different avian species globally. It has been confirmed that MDV cannot be transmitted horizontally from layer hens to eggs but day old chicks become infected in broiler production housing systems from shedders. Several avian species have been grouped based on literature: Free ranging and farm animals and Migratory birds. Chicken red mites have also been identified as carriers of avian pathogens. Free ranging and farm animals consist of turkeys, common pheasant, common grey partridge and European quail. Migratory birds that have been confirmed positive are ducks and white footed geese. Although these birds become infected and are carriers, what has not been identified is their role in viral virulence factors as well as ability to infect broiler production system. Furthermore, the presence of chicken red mites has yet to be confirmed in broiler production system which could contribute to viral persistence.

MDV infection and its global presence could also be a product of natural reservoirs located in backyard and migratory birds (Figure 4) such as Common teal, White-fronted goose, Pintail, European wigeon, and Mallard [48] and more recently in Roulroul partridges [49]. The presence of MDV in wild and migratory birds has been well documented since the early 1980. So it is of no great surprise that new reports emerge as these viruses cause disease in newly identified host. Further monitoring is required to understand the importance of wild birds as reservoirs along migratory routes for pathogenic serotypes.

### Infection of immune cells

Based on the current model of MDV pathogenesis, phagocytic cells such as macrophages and dendritic cells in the respiratory tract become infected either directly or after an initial round of viral replication. The virus can also replicate in macrophages and induce cytolysis in the infected phagocytic cells as demonstrated by high levels of cell death in splenic macrophages expressing three herpesvirus kinetic classes: ICP4 (immediate early), pp38 (early) and gB (late). The level of infection in macrophages isolated from MDV-infected birds depends on the virulence of MDV. The in vivo results demonstrate that, at 4–6 dpi, more virulent viruses (C12/130) induce 3–10 times higher percentages of pp38+ macrophages compared to that infected with less virulent MDV. The results also show that pp38+ macrophages are prone to cell death. Interestingly, MDV antigens could only be detected in MDV infected macrophages, but not in uninfected macrophages that have phagocytosed MDV infected cells [20]. Consistent with this view, Abdul-Careem et al. [17] have shown an up-regulation in nitric oxide synthase (NOS) gene in lung macrophages of MDV infected chickens. This results in production of nitric oxide (NO) reported to have anti-microbial activity against many viruses including MDV for both in vivo and in vitro conditions [20]. Likewise, a similar distribution profiles of MDV antigen have been observed in MDV infected B lymphocytes during the early cytolytic phase. Replication of MDV in the lung induces host innate immune response as demonstrated by up-regulation of Toll-like receptors (e.g. TLR 3 and TLR 7), pro-inflammatory cytokines (e.g. IL-1β and IL-8) and iNOS genes as well as infiltration of macrophages [17]. However, mucosal immune response to MDV in the lungs is ineffective in controlling virus replication [17, 20, 25].

Generally, the infection of mammalian immune cells such as macrophages and B cells with herpes viruses down-regulates the expression of both MHC class I and II molecules consequently, evading hosts’ cell mediated immunity [50]. It has been shown that MDV infection down-regulates surface expression of MHC (B complex) class I (BF) glycoproteins during active but not latent infection of chicken cells [51]. Further studies demonstrated that MDV012 and MDV pUL.49.5 genes (Table 1) are involved in down-regulation of MHC class I molecules by interfering with transporter associated with antigen processing (TAP) function. However, the effects of MHC class I down-regulation in the pathogenesis of the disease is unknown and recombinant viruses lacking the cytoplasmic tail of pUL49.5 exhibit almost similar pathogenicity as wild type virus in both the resistant and susceptible chicken lines [52, 53]. NK cells and more importantly cytotoxic T lymphocytes (CTL) which monitor cell surface MHC class I molecules and viral peptide complex respectively may play a major role in host defence against infection. Contrary to MHC class I, MDV infection up-regulates MHC class II molecules on chicken APC’s such as macrophages [54, 55], and this may contribute to virus spread within the infected host by increasing the interaction between infected macrophages and activated CD4+ T cells. Since MDV requires cell-to-cell contact for virus spread and productive infection of T cells and other immune cells, this up-regulation of MHC class II molecules might, in fact, be essential in the pathogenesis of MD within the infected host by increasing the interaction between infected macrophages and activated CD4+ T cells [51, 54]. HIV infection can also induce activation of innate response and up-regulation of MHC class II molecules which can lead to immuno-pathology [56, 57]. It is still unclear whether MHC class II up-regulation observed after MDV infection can induce immunopathology which is clearly manifested in MDV pathogenesis. In the infected cells, MDV virus expresses a viral antigen with high amino acid sequence homology to CXCL13, termed viral IL-8 (vIL-8). CXCL13 exerts its chemotactic effects by interacting with chemokine receptor CXCR5 and is a major regulator trafficking for B cells and subsets of T cells. This viral chemokine has the ability to recruit immune cells to the site of infection [23]. The deletion of vIL-8 from open reading frames severely affects MDV pathogenesis and tumour incidence [22, 23, 58]. vIL-8 induces chemotaxis of B cells and regulatory T cells (CD4+ CD25+ T cells), and these cells are targets for both lytic and latently infected cells, thus demonstrating a virus specific tropism [22]. Within 24 h after infection, the virus can be detected in the bursa of Fabricius, spleen, and thymus [14, 15]. It is believed that macrophages and dendritic cells can disseminate the virus from the lungs to B cells and CD4+ T cells in these lymphoid organs. Splenic B cells play an important role in the replication of MDV, and the high titre of circulating virus is due to the replication of the virus within B cells, but in the absence of B cells, the virus replicates in other immune cells and MD can still be developed. In the cytolytic phase of the disease, large number of splenic B cells undergoes apoptosis and cell death. Depletion of B cells and CD4+ T lymphocytes in lymphoid tissues, such as cecal tonsils (CTs), of susceptible chicken lines within 5 dpi may contribute to immunosuppression observed in late cytolytic phase of the disease. The depletion of B and T cells in the CTs of the resistant line was minimal at 5 dpi, which also recovered by 21 dpi [59]. Virus replication in B and T cells reaches its peak between 3 and 7 dpi. B lymphocytes constitute the majority (around 90%) of cytolytically infected (MDV-antigen positive) cells, while CD4+ and CD8+ T lymphocytes represent only 3 and 8%, respectively [60, 61]. MDV can be transmitted directly to T cells from the infected macrophages or dendritic cells; however, the transfer of virus from B cells to T cells is also conceivable.

**Table 1:** **MDV genes and their respective products involved in immune modulation and pathogenesis**

MDV gene (protein)	Function	Antigenic potential	Infection stage	Reference
MDV003/078 (vIL-8)	Viral chemotactic (CXCL-) protein involved in recruiting immune systems cells to site of viral replication	–	Lytic replication	[23, 58]
MDV010 (vLIP)	Shares homology to host lipase enzyme and forms covalent bonds with lipids	–	Lytic replication	[136]
MDV011/012	Immune evasion protein that down regulates cell surface MHC I expression	–	Lytic replication	[52]
MDV012 ORF012	Phosphoprotein required for viral growth both in vivo and in vitro	–		[137]
MDV040 (gB)	*Hypothetical function*: Virion membrane protein that heterodimerizes with other glycoproteins facilitating viral fusion with host membrane	Yes	Lytic replication	[26, 27]
MDV052/053 UL39/40 (RR)	Viral Ribonucleotide Reductase (RR) is essential for replication both in vivo and in vitro	–	Lytic replication	[138]
MDV064 (U_L_49.5)	Partially reduces MHC I expression by interacting with TAP protein	–	Lytic replication	[53]
MDV073 (pp38)	Early protein expressed during cytolytic infection and phosphorylated by U_s_3p	Yes	Lytic replication	[26–29]
MDV092 (U_s_3p)	Serine/threonine protein kinase that phosphorylates pp38	–	Lytic replication	[139]
MDV084/100 (ICP4)	Viral gene transactivation function	Yes	Lytic replication	[26, 27]
MDV001a (vTR)	Required for integration of viral genome into host DNA for immune evasion, neoplastic transformation and viraemia	–	Latency	[140]
MDV006 (pp14)	Neurovirulence factor required for PNS neuropathy (acute or transient paralysis)	–	Latency	[141]
MDV062 (VP22)	Tegument protein essential for viral replication and modulates host cell cycle	–	Latency	[142]
MDV057 (gC/UL44)	Type 1 transmembrane protein required for horizontal transmission/shedding from feather follicle epithelium	–	Feather follicle shedding	[143]
MDV005/076 (MEQ)	Viral oncogenic protein involved in T cell neoplastic transformation which forms homodimers and heterodimers with specific intracellular signalling proteins that modulates host cell cycle	Yes	Neoplastic transformation	[26, 27, 35]
MDV029 (pUL17)	Co-localizes with VP5 and VP13/14 tegument protein and essential for in vivo viral growth, capsid maturation and DNA packaging	–	Neoplastic transformation	[144]

Cytolytic infection of B and T cells is semi-productive, which is defined by their inability to express certain viral structural components. No cell-free virus is produced by the infected T and B lymphocytes and only non-enveloped intra-nuclear particles are detected [5]. The precise mechanism as to how in vivo MDV spreads from cell to cell has not been elucidated. However, it is assumed that MDV glycoproteins (g) B, gC and gD, similarly observed in most herpes viruses, are likely to interact with host’s cell surface receptors; thereby, forming an intracellular bridge between infected and uninfected cells which might, in turn, contribute to cell associated viral spread in MD. Similarly, co-existence of MDV gH and gL have been reported to be vital for GaHV-2 cell to cell viral spread. The primary peptide of gL has a high affinity for specific region of gH, and therefore binds to it giving rise to a complex, hetero-oligomer structure that anchors itself onto the cell surface of infected host cell, promoting MDV proteins (gp) cell surface expression [62]. In support, Schumacher et al. [63] demonstrated that deletions of gE (20DeltagE) or gI (20DeltagI) were essential in restricting viral spread and plaque formation, although viral replication was not abolished. Transfecting gE or gI did not support viral spread indicating that gE and gI could work in synergistic manner to aid viral spread. Furthermore, it is possible that MDV mainly replicates within B and T cells by mitosis of infected cells rather than production of virions. Thus, MDV does not require expressing all the viral genes to replicate in vivo. By not producing all the MDV viral antigens that may be highly immunogenic, the virus can escape immune control. This notion is supported by the fact that the resting T cells, with low proliferative abilities, are less susceptible to MDV infections than the activated lymphocytes. T cell activation may increase the expression of surface molecules that is engaged in virus entry, thus enhancing the occurrence of MDV infection in these cells.

Similar to MDV, the infection of human CD4+ T cells by human T cell leukaemia virus type 1 (HTLV-1) is increased in activated CD4+ T cells. This is reflected on up-regulation of heparan sulfate proteoglycans (HSPGs), a receptor for HTLV-1, on activated CD4+ T cells [64]. No specific receptor for MDV entry into chicken CD4+ T cells has yet been identified. It is conceivable that MDV infection induces cytolysis/inflammatory responses and T cell activation, which leads to T cell infection and viral replication via mitosis by passing the virus to daughter lymphocytes.

The underlying genetic variation within different chicken lines may play a key role in the pathogenesis and prognosis of the MDV infection. The percentages of T cells that become cytolytically infected in the lymphoid organs are less than 2% in MD-susceptible chickens and 0.2% in the resistant chickens [5, 60]. In support, Omar et al. [26] have demonstrated evidences of T cell mediated immune responses against the MDV antigen, *ICP4*, only in resistant (B21) chicken lines; but not in susceptible (B19) ones. In contrast, both B21 and B19 chicken lines have been reported to show T cell immune responses against MDV antigens: *gB*, *Meq* and *pp38*. Nevertheless, these studies suggest that the role of T cell mediated immunity, and differences in T cell receptor repertoire generation in conjuncture with other inherent genetic resistance mechanism against MD cannot be ruled out. In latent phase of infection, MDV antigens cannot be detected in the lymphoid tissues and there is no production of infectious viruses. It is difficult to distinguish latent infection from transformation phase, as both represent non-productive infections. Following in vitro reactivation of MDV from virus antigen-negative lymphocytes, the majority of latently infected lymphocytes were identified as T cells with only 3% being B cells [5]. *Meq* antigen from MDV plays a crucial role in maintaining latency by blocking apoptosis of latently infected CD4+ T cells. One of the pronounced differences in gene expression profile between MDV-resistant and susceptible chicken lines after MDV infection are genes that are associated with apoptosis [65]. In addition to *Meq*, it has been shown that microRNA miR-M3, an MDV-encoded miRNA, abrogates apoptosis by directly targeting Smad2, a critical component in the transforming growth factor (TGF)-β [66], providing an environment beneficial for latency and oncogenesis. miR-M4, an ortholog of the oncogenic miR-155, was shown to have a direct effect on inducing MDV-induced T cell lymphoma, as viruses deleted in miR-M4 or having mutations in the seed region failed to induce lymphoma [67]. Viral miR-M4 exerts its effects by reducing the levels of latent TGF-β binding protein-1, which is involved in the maturation of TGF-β. This leads to a reduction in the levels of active Smad2/3 and release of the inhibition of the c-Myc promoter, resulting in a rise in c-Myc transcription. The production of viral protein *Meq* allows the formation of complexes with c-Myc [68], which is associated with transformation. In the susceptible chickens, a second wave of semi-productive infection and cytolysis are observed between 14 and 21 dpi [14]. This late cytolytic phase is associated with immunosuppression, atrophy of lymphoid tissues such as thymus, bursa of fabricius, cecal tonsils and infiltration of mononuclear cells and heterophils [14, 69]. Virus is probably transferred to the skin by latently infected CD4+ T cells and infects skin and feather follicles in a yet unknown mechanism. Syncytia formation in skin epithelium may be a product of viral protein which facilitates and pools greater resources for viral replication. The involvement of other immune system cells such as macrophages and dendritic cells to transport the virus to the skin cannot be excluded. Replication of MDV starts at 7 dpi, well before tumour development. Therefore, it is possible that early infected CD4+ T cells, but not necessarily latent or transformed CD4+ T cells, transport the virus into the skin at this time.

## The immune response to MDV

### Innate immunity

While effective immunity against human *alphaherpesviruses* relies on both innate and adaptive mechanisms, the innate immune response has been shown to be of paramount importance [70]. Less is known about the role of innate immunity in the control of MDV in chickens.

#### Interferons

Type I IFNs belong to a family of cytokines that attracted much attention owing to their protective role against viral infection. IFNs are widely expressed cytokines that possess strong antiviral and immunomodulatory properties. The IFN family can be classified into three main types of cytokines—type I, type II and type III IFNs. IFN-α and IFN-β belongs to type I IFN family, while the type II IFN family includes only one cytokine: IFN-γ, which also exhibits antiviral activities [71]. The third type of IFNs is the IFN-λ family. In mammals, plasmacytoid dendritic cells (pDCs), monocytes, epithelial cells and fibroblasts are the main producers of type I IFNs [72], while type II IFNs are predominantly produced by NK cells and activated T cells. In spite of the fact that chicken type I IFNs are shown to inhibit viral infection both in vivo and in vitro, chicken pDCs have not been identified.

Chickens become infected with MDV via the respiratory system by inhaling infected dust. MDV is taken up by phagocytic cells such as macrophages or dendritic cells within the respiratory system. Chicken lung has a different anatomical structure than the mammalian counterpart consisting of air sacs; and due to narrower pulmonary capillaries than in mammals there are fewer airway resident macrophages [73]. Therefore, it is likely that MDV has to cross lung epithelial lining before being transported by phagocytic cells to lymphoid tissues. In the respiratory system, the virus can be recognized by TLRs, such as TLR21 (recognizing unmethylated CpG DNA), leading to the initiation of protein signalling cascaded which stimulates the expression of type I interferons (α and β), shown to be involved in antiviral defence. In fact, an increase in the expression of TLR receptors in the lungs of MDV-infected birds [36], IFN-α expression in the blood of susceptible chickens [74] and interferon regulatory factors (IRF)-1 and IRF-3 in MDV-infected chicken embryonic fibroblasts cells (CEF) have been reported [75, 76]. The role of IFNs in the control of MDV replication has been confirmed in an in vitro model showing that IFNs reduces plaque formation and expression of *PP38* and *gB* in the infected cells [77]. The protective role of IFNs is also indicated by the results demonstrating the differential expression patters of IRF-3 and IFN-β genes in resistant and susceptible chicken lines [78]. Similarly, oral administration of IFN-α are shown to reduce MDV viral replication in vivo [79].

In addition to their direct effects on viral replication, type I interferons may also activate other immune system cells such as natural killer (NK) cells and increases their cytotoxic function [80]. However, it has been suggested that chicken NK cell cytotoxicity is not increased after oral administration of recombinant chicken IFN-α or inoculation of recombinant MDV expressing chicken IFN-α (rMDV-cIFN-α). In chickens inoculated with rMDV-cIFN-α, NK cell cytotoxicity was not enhanced over control chickens at 4 and 7 dpi. Furthermore, at 4 dpi, chickens inoculated with R2/23 actually had decreased NK cell cytotoxic activity. Therefore, it is concluded that ChIFN-α, given at high doses orally in the drinking water or via expression in a recombinant MDV vector, does not increase NK cell cytotoxicity as originally hypothesized [79]. The suppression of immune responses by oral administration of IFN-α is not a novel phenomenon as similar observations are reported in murine model where, bone marrow functions were suppressed by administration of murine IFN-α orally or subcutaneously [81]. In fact, it has been shown that type I IFNs modulate the function of both innate and adaptive immune cells including DCs and T cells in the gut and suppress the intestinal inflammation. Taken together, it is believed that type I IFN response by MDV-infected cells promotes the activation of immune cells and inhibits MDV replication and dissemination. Considering the complexity of MDV and co-evolution of the virus with the host’s type I IFNs response, it is very likely that there is a complex relationship between MDV and host response. The exact role of type I IFNs in the pathogenesis of MDV in chickens is poorly understood due to lack of immunological reagents, complexity of MDV infection and the cell-type specific effect of type I IFNs. However, it is possible that type I IFNs may be involved in promoting latency infection of MDV as has been observed in other *alphaherpesviruses* [82].

The role of type II IFNs, IFN-γ, is discussed in more detail in adaptive immunity section. IFN-γ is induced during MDV infection and shown to have inhibitory effects on MDV replication by inducing nitric oxide production [77] and the administration of IFN-γ with MDV vaccine positively influenced vaccine-induced protective immunity in vivo [83]. There is no information on the role of type III IFNs on viral infections in chickens.

#### Macrophages and dendritic cells

There is very little information on the type and function of antigen presenting cells (APCs) involved in the initiation of immune responses against MDV in the respiratory system of chickens. However, it is believed that chicken professional APC’s such as macrophages and dendritic cells play an important role in the development of adaptive immunity against MDV. In mammals, it has been shown that dendritic cells play a crucial role in linking innate to adaptive immunity. The type of dendritic cells and the type of maturation induced by different stimuli influences the immunological outcome, such as differentiation of Th1 vs. Th2 type T cells. There is very limited information on the role of dendritic cells in MDV immunity, however, it is postulated that these cells could be involved in the initiation of innate and adaptive immunity against MDV. In addition to their ability to present MDV antigens in association with MHC class I and II molecules to initiate adaptive immunity, macrophages can also be directly involved in inhibition of MDV replication and development of MD. Macrophages isolated from B21 chickens have higher phagocytic activity than B19 chickens to MDV [84]. Moreover, macrophages obtained from MDV-infected chickens inhibited viral replication in vitro more efficiently than macrophages isolated from non-infected chickens [85]. Further confirmation on the role of macrophages in MDV infection is obtained from the results demonstrating that depletion of macrophages from splenocytes increases MDV replication [84], while stimulating macrophages in vivo reduces the incidence of MD [86]. Taken together, the evidence presented here from several studies confirms that macrophages play a pivotal role in control of MDV replication and MDV-derived tumour incidence. One of the mechanisms involved in the inhibitory function of macrophages on MD is their ability to produce inducible nitric oxide (iNOS). The production of NO has been reported in the spleen, brain and lungs of MDV-infected chickens [13, 77, 87, 88] and NO is shown to inhibit MDV replication with highest level of NO production detected in serum and spleen of resistant chickens compared to susceptible chickens [77, 89]. Further experiments demonstrated that the inhibition of iNOS in chickens increases viral load, suggesting that NO plays an important role in the control of MDV replication in vivo [77]. Another function of macrophages is their ability to kill tumour cells and it is believed that activated chicken macrophages have the ability to lyse MDV-derived tumour cells in vitro [90]. In contrast to activated and fully functional macrophages, tumour associated macrophages (TAMS) represent key regulators of the complex interplay between the immune response and cancer. TAMS produce tumour growth promoting factors and induce immunosuppression by releasing immuno-modulatory factors [91]. Macrophages isolated from tumour tissues of MDV-infected chickens demonstrate similar functional abilities as TAMS and have been shown to suppress T cell proliferation in vitro. The development of immuno-regulatory macrophages in MDV-infected chickens correlates with transient immunosuppression observed during primary cytolytic phase of infection [92], suggesting that TAMS may be involved in MDV-induced immuno-suppression.

#### Natural killer (NK) cells

NK cells represent important effectors of the innate immunity and can respond to stimuli and produce anti-viral cytokines such as IFN-γ. In addition, these cells have ability to recognize virus- infected cells/tumour cells via ligation of cell death receptors and the release of granules. NK cells from MDV-resistant chickens have higher cytotoxic capability than the MDV-susceptible chickens, suggesting that these cells may be involved in determining resistance to MDV. This hypothesis is also supported by the results demonstrating that MDV-infected chickens have higher NK cell activity than the cells isolated from the non-infected birds and; this activity lasted longer in the resistant chickens than the susceptible chickens [93]. The exact role of NK cells in providing vaccine-induced protection against MDV is still unknown, however, there are some evidences demonstrating that vaccination against MDV increases the functional abilities of NK cells. This may explain how MDV vaccine can provide protection in vaccinated chicks as early as 3 days post-vaccination [93]. NK cells also play a fundamental role as antitumor senses through downregulation of cell surface markers such as MHC I. A notable characteristic of herpes viral infection and specifically MDV is down regulation of MHC I cell surface translocation. Further to that, anti-tumor activity has not yet been demonstrated in an MDV resistance model independent of genetic factors that predispose resistance to neoplastic transformation of CD4^+^ T cells. Taken together, it has been suggested that NK cells may play an important role in controlling MDV infection [93–95]. Using recently identified markers such as CD56 and CHIR-AB1 [96] for identification of chicken NK cells, researchers will be able to elucidate the role of these important cells in providing protection against MDV.

### Adaptive immunity

The key components of adaptive immunity are B and T lymphocytes which specifically recognize antigens and generate memory response. B cells are involved in humoral immune response, whereas T cells are involved in cell-mediated immune response.

#### Humoral immunity

As MDV is a cell-associated herpes virus and is strictly intracellular, antibodies should not have a major role in the protective immunity against MDV infection. However, antibodies against several MDV glycoproteins including gB, gE, gI have been detected in MDV-infected birds [97, 98]. The role of these antibodies in providing protective immunity against MDV has not been clarified. However, there is some evidence suggesting that anti-gB neutralizing antibody may have a protective role against MDV, perhaps via blocking virus entry into the host cells or antibody dependent cell-mediated cytotoxicity (ADCC) of infected cells. The role of antibody response in the control of MDV infection is also confirmed by the fact that the presence of maternal antibody delays the development of clinical signs and tumour. However, the presence of maternal antibody can interfere with live replicating vaccines against MDV by neutralizing the vaccinal virus [5]. Therefore vertical transmission of immunological factors such as maternal antibodies can be a limiting factor for both generations of protective immunity as well as delaying immune responsiveness to infection challenge models of offspring from vaccinated layers. Maternal antibody and interference with vaccination has been reported in both human and veterinary medicine regardless of the types of vaccine formulations used. It has been shown that while maternal antibody can interfere with antibody response, vaccine-induced cell-mediated immune responses are largely unaffected. This has been confirmed in humans, murine models and farmed animals [99–104]. With regard to the inhibitory effect of maternal antibody response, new strategies have been developed to overcome the inhibitory effects of maternal antibody on vaccine-induced antibody response. For example, it has been shown that the induction of type I interferon in vivo strongly stimulates B cell responses and restores antibody levels after immunization in the presence of maternal antibodies. One way of inducing high levels of type I interferon is the combined use of TLR-3 and TLR-9 agonists as adjuvants for immunization [102]. Therefore, it should be possible to overcome the neutralizing effects of maternal antibody against MDV in novel vaccination strategies.

#### Cell-mediated immunity

Antibody and cell mediated immunity is involved in the control of infection with highly cell-associated human *alphaherpesviruses* such as VZV. Both CD4+ and CD8+ effector and memory T cells are shown to be essential for recovery from VZV and maintaining the latent stage of infection in the subclinical state. Generally, it is believed that (a) broad (response to several epitopes), (b) durable (memory response), and (c) multi-functional (capable of producing several Th1 type cytokines/kill virus infected cells) T cell response is associated with the control and resolution of viral infections. MDV is also a highly cell-associated alphaherpesvirus, and thus it is postulated that cell-mediated immunity is crucial for the vaccine induced protection [105, 106]. Since MDV exists in cell associated forms inside the host, except in feather follicles, T cell mediated immunity is thought to be more important than the antibody mediated immunity in the control of the disease in chickens [107]. Studies have shown evidences demonstrating the presence of CD8+ T cells against MDV antigens such as: *gB*, *Meq*, *pp38* and *ICP4*. However, the role of CTL in conferring long term immunity, generation of memory cells, in genetically resistant chickens is unknown [36] and the role of cell-mediated immunity in vaccine-induced protection has not been determined. No cytotoxic response against *ICP22* and a weak cytotoxicity against *Meq* were detected in MDV-infected chickens [5, 26]. Other studies confirmed the presence of anti-*gB* and anti-*gI* TCRαβ1+ CD8+ T cells with cytotoxic abilities in MDV-infected birds [108].

The presence of herpes virus specific CD8+ T cell response coincides with protection from cytomegalovirus (CMV) infection and adoptive cell transfer of CD8+ T cells provide protection in animal models. The importance of CD4+ T cells in providing protection against herpes virus infection was also demonstrated by CD4+ T cell depletion experiments in animal models. There was an inverse correlation between the number of virus-specific CD4+ T cells and prolonged shedding [105, 106]. Therefore, it is very likely that both CD8+ and CD4+ T cells are involved in the control of MDV replication. This notion is confirmed with the early studies showing that T cells are crucial for the control of tumour growth in HVT-vaccinated chickens. The importance of T cells in the control of tumour growth was confirmed by demonstrating that HVT vaccinated birds treated with cyclosporine, a drug shown to inhibit T cell function, develop MDV-lymphoma [109, 110]. However, later studies suggested that T cells are only involved in the control of viral replication but not essential for the control of tumour growth [111]. Further studies are required to confirm the exact role of CD4+ and CD8+ T cells in the control of MDV replication and tumour growth. The availability of CD4+ and CD8+ knockout chicken will provide valuable tools to study the role of these cells in vaccine-induced protective immunity against viral replication and tumour growth. MHC-determined resistance to MDV in MHC:B21/B21 birds indirectly confirms the role of T cells in control of tumour development. For MHC class I molecules, the relative level of expression varies between different MHC haplotypes and reflects the consensus hierarchy of response by different MHC haplotypes with the most susceptible chickens (MHC B19/B19) having the highest expression while the most resistant chickens (MHC: B21/B21) expressing the lowest level [112]. It is believed that the differences in cell surface expression level ensure the development of optimal peripheral T cell responses against MDV. The detection of MDV-specific CD8+ T cells with cytotoxic ability to recognize target cells expressing MDV antigen is technically challenging. However, Schat and Markowski-Grimsrud developed a heterologous system to investigate cytotoxic T cell response to MDV using reticuloendothelial virus-transformed cells stably transfected with specific MDV genes [99]. The cell lines derived from resistant (MHC: B21/B21) and susceptible (MHC: B19/B19) birds were generated and antigen-specific cytotoxicity were analysed in vitro. The cytotoxicity was low compared to mammalian cytotoxic T lymphocyte (CTL) assays [5]. In these studies, relatively a more potent cytotoxicity was detected against target cells expressing pp38 and gB antigens. No cytotoxic response against ICP22 and a weak cytotoxicity against *Meq* were detected in MDV-infected chickens [5, 26]. Other studies confirmed the presence of anti-gB and gI TCRαβ1+ CD8+ T cells with cytotoxic abilities in MDV-infected birds [108]. Vaccination of chickens with a recombinant Fowl Pox Virus (FPV) expressing these two glycoproteins have been reported to induce protective immunity when challenged with MDV and shown to induce anti-MDV neutralizing antibodies [113, 114]. Recombinant FPV expressing *gB* was later shown to induce cytotoxic T cell response recognizing target cells expressing *gB* [115]. These data support the importance of anti-MDV CD8+ T cells responses in the control of the disease, however, the role of MDV-specific CD8+ T cell responses in the control of viral infection or tumour growth in birds immunized with HVT or CVI988-Rispens is still unknown. In addition, there is very little information on the magnitude and quality of MDV-specific T cells responses in resistant vs. susceptible chickens. The only available information suggest that anti-*ICP4* CD8+ T cell responses is only detected in the resistant chickens [26], while anti-*gE* CD8+ T cell response is only detected in the susceptible chickens. These results suggest that the response to *ICP4*, an immediate early MDV gene product, but not to *gE* may be an important factor in genetic resistant to MDV. It has been shown that CTL responses characterized by release perforin and granzymes can be induced against MDV antigens such as *PP38*, *Meq* and *ICP4* [36]. Moreover, there is still no information on the presence of MDV-specific CD4+ T cell responses and whether transformed CD4+ T cells have antigen specificity to MDV antigens. Further studies are required to identify CD4+ and CD8+ T cell epitopes within MDV antigens and determine their MHC restriction. Moreover, it is important to examine the quality, broadness, durability and magnitude of the CD4+ and CD8+ T responses in MDV infected or vaccinated chickens and determined the correlates between T cell responses and protection.

In addition to T cell responses against MDV viral antigens, it is possible that T cells may also recognize self-antigens expressed by tumour cells. However, the nature of these self-antigens and their relevance to immunity and inhibition of tumour growth in birds is still unknown. A few decades ago, Marek’s disease tumour-associated surface antigens (MATSA) was identified in the MDV-transformed T cells [116]. However, it was later discovered that activated T cells can also express MATSA antigens, and this molecule is not solely expressed on transformed T cells [117]. Several of these MATSA are found to be activation associated lymphocyte antigens, and one of them is identified as CD30 molecule [47]. Meanwhile CD30, a co-stimulatory molecule, has been shown to have pleiotropic effects on human T cell activation, apoptosis, effector function (cytotoxicity) and regulating T cell trafficking/migration [118]. It has a fundamental role across all T cell lineages such as CD4+ , CD8+ and Th17 cells [119] and may influence T cell interaction; suppressive action in the tissue microenvironment.

**Figure 5:**
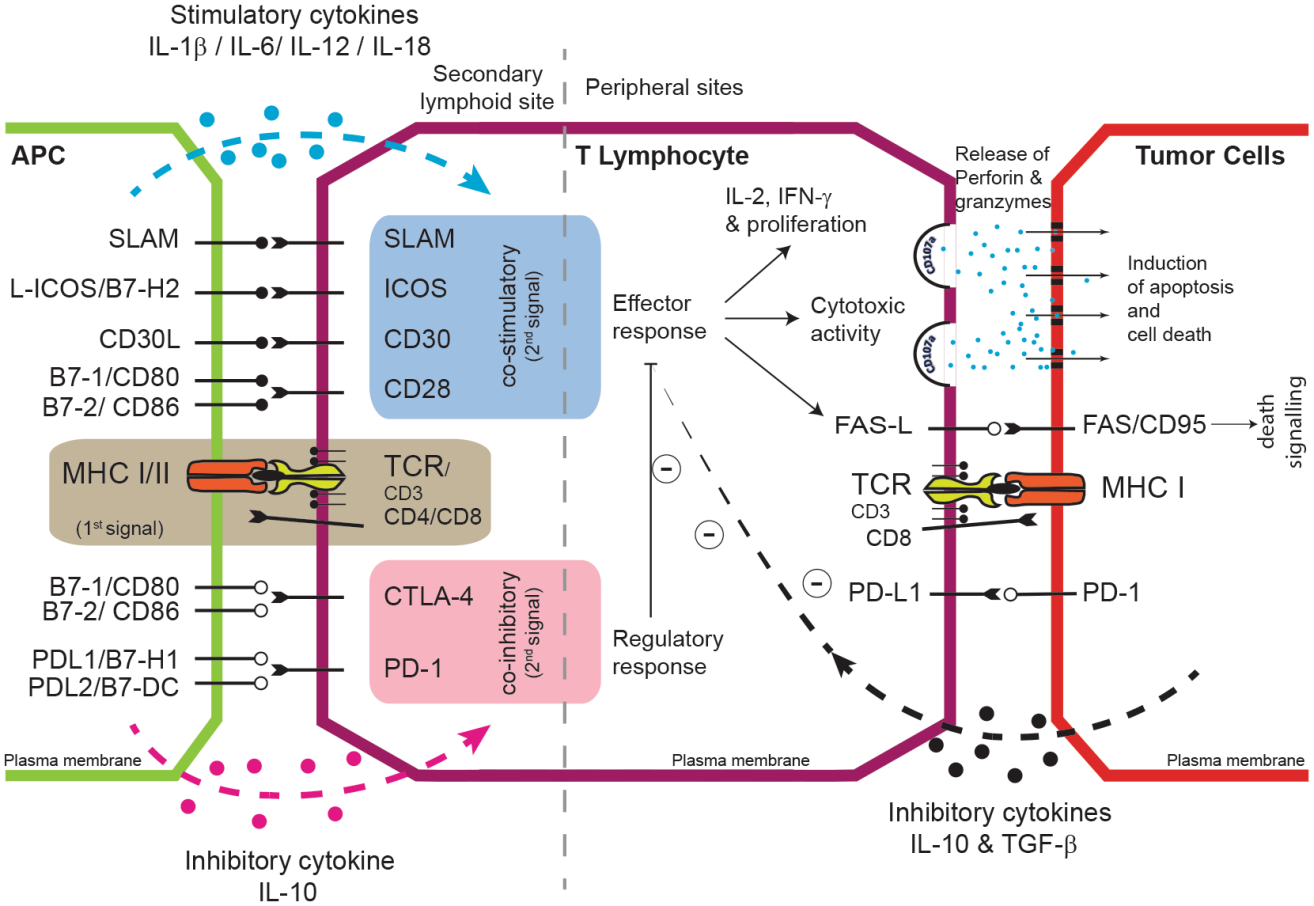
**T cell activation and tolerance by tumour cells/antigen presenting cells.** Inhibitory or stimulatory molecules expressed on the surface of antigen presenting cells (APC) or tumour cells regulate T cell function. Moreover, stimulatory or inhibitory cytokines may drive the generation of different T cell populations (e.g. Th1, Th17, Treg etc.) with diverse functional properties. Signal one is provided to the T cell receptor of T cells by presentation of antigens via MHC class I or II molecules expressed by APCs or tumour cells, while the signal 2 is provided by co-stimulatory molecules such as B7 family. Co-stimulatory signals induce the generation of effector T cells, which can recognize and lyse target cells or produce cytokines such as IFN-γ, involved in the control of tumour growth. In contrast, inhibitory molecules deliver negative signals and suppressing the effector T cell function, and induce T cell anergy or exhaustion.

Immunomodulatory processes that influence CTL functional abilities in relation to persistent viral infection and tumorigenesis may be mediated via interaction of inhibitory receptors such as CTLA4, programmed death-1 (PD-1) and its respective ligand programmed death ligand-1 (PD-L1). The evidence suggests that MDV infection up-regulate the expression of CTLA-4, PD1 and PD-L1 in chicken immune system cells. The expression level of PD-1 was increased in chickens at the early cytolytic phase of the MDV infection, while the PD-L1 expression level was increased at the latent phase. In addition, the expression levels of PD-1 and PD-L1 were increased at tumor lesions found in MDV-infected chickens [120, 121]. Furthermore, T cell responsiveness may be affected by chronic antigen stimulation leading to exhaustion thus altering phenotypic characteristics such as PD-1 expression also observed during in vitro MDV infection. A combination of reduced MHC-I and increased PD-1 cell surface translocation provides a platform for highly efficacious immune evasion tactic. CTLA-4, a potent inhibitory receptor, expressed by CD4+ T cells has also be reported during the early cytolytic phase in MDV infected birds [120]. Significant differences in CTLA-4 and PD-1 expression levels, which could result in a delayed immune responsiveness (anergy) and cell death respectively, were reported between resistant and susceptible lines of chickens challenged with MDV. A similar phenotype has been reported in patients infected with human *alpha*-herpes viruses such as VZV whereby CD4+ T cell predominantly express CTLA-4 and PD-1 [122]. Induction of an early immune unresponsiveness (anergy) and cytolysis (4–7 dpi) could well be a hallmark, for *alpha*-herpes viruses, to establish early semi-productive viral replication and disseminated viraemia. Blocking or down regulating CTLA-4 or PD-L1 could be therapeutically significant during early infection. Furthermore, PD-L1 expression results in immune suppression thus can be used as a marker for CD4+ T cell lymphoma. PD-L1 expression in MDV challenged chickens, could limit the quality of T cells immune responsiveness to *Meq*, *pp38*, *ICP4* or *gB* at the immunological synapse (Figure 5). PD-1 and CTLA-4 ligation with its cognate receptor results in cross-pathway interference resulting in inhibition of RAS, ERK 1/2, AKT, JNK and PLCγ phosphorylation altering cell fate; growth/proliferation, effector function and survival. Expression of inhibitory molecules on both antigen presenting cells can lead to the generation of chicken regulatory CD4+ T cells (Treg cells). The role of naturally occurring and peripheral derived Treg cells in modulation of anti-MDV immunity is still unknown. However, it is known that MDV infection up-regulates the expression of inhibitory molecules such as CTLA-4 and expression of inhibitory cytokines such as IL-10. The expression of regulatory molecules such as CTLA-4 and IL-10 on both CD4+ and CD8+ T cells are at 10 and 21 dpi and this effect was more pronounced in the MDV-susceptible chicken lines [123].

## Persistence in the face of vaccination

Vaccination against MD started in the late 1960s using turkey herpesvirus (HVT) which does not induce disease in chickens. The vaccination reduced the incidence of MD by 99% and was the first successful vaccine against naturally occurring virus-induced cancer. Since then MDV has evolved, perhaps due to vaccine induced immune response, and the escape of new mutants from immune pressure. Currently only attenuated MDV strain; CVI988-Rispens is effective in providing protection against the very virulent MDV. Live vaccines are administered either to day-old chicks or to the 18-day-old embryo. MDV vaccine inhibits the development of MDV-induced lymphoma but does not prevent infection and replication of pathogenic strains of MDV. Both vaccination and maternal antibodies against MDV increases viral shedding and onward transmission of hyper-virulent strains of MDV due to the survival of the host without controlling the virus shedding [124]. The failure of current vaccines to induce sterile immunity can be attributed to MDV inducing latency with minimal viral replication and viral protein expression. The ideal MDV vaccine is to control both the disease and viral shedding in the infected birds. Revaccination (prime-boost) with the current cell-associated MDV vaccine (CVI988-Rispens) improve protection against the disease and increases the magnitude of anti-MDV T cell responses as demonstrated by enhancement of anti-MDV neutralizing antibody and proliferation of CD4+ and CD8+ T cells [125]. However, the type and quality of immunity after even revaccination with the current MDV vaccines cannot control virus shedding. Further research is required to understand immune responses required to control MDV shedding as well as the control of the disease. To generate more effective vaccines against MDV, we need to (a) have a better understanding of the type of immunity required for reducing viral shedding/inducing sterile immunity (b) design a vaccine that can induce protective immunity against the virus. It is believed that the quality, broadness, durability and homing of both CD4 and CD8 T cell responses may explain why vaccines against some *alpha*-herpes viruses (VZV) are more effective than vaccines against other *alpha*-herpes viruses (HSV) [126]. As yet there is little information as to whether cell-mediated immunity is induced by the MDV vaccine. The lack of established methodology to assess cell-mediated immunity to MDV vaccine is the major obstacle to have a better understanding of how MDV vaccine actually works. It is very likely that the induction of both innate and adaptive immune responses by vaccine strain is similar to those induced by pathogenic MDV [93, 127]. Cytokine response following oncogenic and vaccine strains of MDV has also been reported and the results demonstrate that very virulent virus (vvMDV), compared with vaccine strain (CVI988/Rispens), induced similar levels of the typical Th1-type cytokine IFN-γ in microglia in vitro, while vvMDV induces higher expression of IL-12 (p40), IL-8, and MIP-1β [128].

It is postulated that vaccine-induced adaptive immunity plays a major role in providing protection against the disease. There are some evidences demonstrating that a number of MDV antigens, including *gB* when administered as recombinant vaccine in a fowl pox vector, are immunogenic and immunity to these antigens confers protection [115]. However, the types and magnitude of vaccine-induced protective immune responses to these antigens are still unknown. Several factors including genetic background of chickens [129], presence or absence of maternal antibodies, virulence of MDV, vaccine dose [130] and concurrent infections with other immunosuppressive pathogens such as CIAV [108] can influence the efficacy of MDV vaccines.

A medley of growth factors and cytokines such as myelomonocytic growth factor (MGF) [131] and IFN-γ [83] have been considered as immuno-modulators and vaccine adjuvants against MDV. The treatment of MDV-susceptible chickens with MGF, a growth and activation factor for monocytes/macrophages, reduced viral load, increased survival rate and reduced tumour incidence after challenge [131]. It is postulated that MGF increases the number of macrophages and their response to stimuli as shown by an increase in NO production by activated macrophages. Similarly, we have recently shown that administering HVT vaccine with a plasmid expressing recombinant chicken IFN-γ enhanced the protective efficacy of the vaccine against MDV and reduced viral load and tumour incidence [83]. IL-18 has been shown to stimulate IFN-γ production from CD4+ T cells and can also indirectly stimulate CD8+ T cell proliferation [132]. In addition, IFN-γ can enhance the development of Th1 type responses which is known to be important in the control of viral infections as well as activating innate immune cells such as macrophages and NK cells. Although not explored in chickens, classical activation of IFN-γ results in interferon stimulatory gene (ISG)-1 up regulation, which could have an additive antiviral effect. Both type I and type II interferons have been tested in various clinical settings but the whole gamete of IFN-regulated signalling pathways have yet to be elucidated. Ligands for TLRs are used as adjuvants that stimulate immune responses, leading to protection against infectious diseases. We have demonstrated that the administration of TLR3 [133], TLR4 and TLR21 ligands [134] reduces the incidence of tumours and MDV genome copy numbers in the infected birds. The importance of TLR in providing protection against tumour development could be extrapolated from the results demonstrating significantly higher basal expression levels of TLR3 and TLR7 in uninfected chicken cells isolated from resistant chicken lines compared to the susceptible lines [135].

## Conclusions

Our understanding of innate and adaptive immunity to MDV infection is continuously improving. This growing knowledge can be further enhanced by our understanding of immunity to human *alphaherpesviruses* and host-pathogen interaction. However, there are many differences between the human and avian immune system as well as different characteristics between MDV and human *alphaherpesviruses* (VZV, HSV1 and 2). For example, MDV is the only *alphaherpesviruses* that can modulate the chemokine network by molecular mimicry of a host protein and thus recruit, infect and transform CD4+ T cells. In terms of vaccine development, the developments of potent cell-free vaccines that can inhibit infection as well as the disease are paramount to research in this area.
